# Experimental Analysis of Passenger Comfort with Variable Preloaded Rear Springs on a Low-Cylinder Motorcycle

**DOI:** 10.3390/s23136204

**Published:** 2023-07-06

**Authors:** Ronald Jiménez-Canoas, David Alejandro Collazos-Burbano, José Isidro García-Melo, Jorge Lopera

**Affiliations:** 1 School of Mechanical Engineering, Universidad del Valle, Calle 13 No. 100-00, Cali 760032, Valle del Cauca, Colombia; 2 Centre for Bioinformatics and Photonics—CIBioFi, Universidad del Valle, Calle 13 No. 100-00, Cali 760032, Valle del Cauca, Colombia; 3 Grupo de Investigación Logística y Producción, Industrial Engineering School, Universidad del Valle, Calle 13 No. 100-00, Cali 760032, Valle del Cauca, Colombia; 4 PACDE SAS, Pacific+, Av 9a #21n-97, Cali 760043, Valle del Cauca, Colombia

**Keywords:** comfort, ISO 2631, low-cost motorcycle, public health policies, rear suspension, whole body vibration

## Abstract

The present study contains an experimental analysis of the vibratory response in a low-cylinder engine motorcycle at varying suspension preloads. Three different speed bumps of varying heights were used to subject the motorcycle to different vibrations. The analysis was carried out in three domains: time, frequency, and time-frequency. A triaxial accelerometer was used to measure the vibrations at the seat of the vehicle. The results indicated that the suspension system became more differentiated as the height of the bumps increased. However, for lower bumps, the action of the three spring preloads studied was quite similar. Quantitatively, only the higher bump showed a significant difference between the set preloads. The spectral distribution revealed that the frequency of interest was below 20 Hz for all the studied cases, which is in the same range of human body natural frequencies. The findings of this research can be utilized to enhance the design of low-cost motorcycles, thereby improving the safety and comfort of their drivers and passengers. This study constitutes a significant step towards developing an affordable system capable of gathering sufficient data to support the creation of evidence-based public health policies and propose new transport industry standards based on field measurements.

## 1. Introduction

Motorcycles have become one of the most used vehicles worldwide for various purposes, such as delivery, mobility, policing, sports, and postmen activities. Indeed, recent data on the Latin American motorcycle market indicate that sales have grown by more than 10.7% [1]. The reasons for the extensive use of these vehicles vary between low- and high-income countries. In developing countries, like those in Latin America, motorcycles with low-cylinder engines are primarily used for daily commuting to and from work or school due to increasing fuel costs and heavy traffic congestion, offering time and cost savings [2]. These vehicles offer numerous advantages, including their small size, which makes them easier to park, and their low maintenance and insurance costs. In contrast, motorcycles in developed countries are often more advanced (with higher cylinder engines), generally do not carry passengers, and are mostly used for recreational purposes, such as extreme sports and road trips [3]. Typically, the higher the human development index of a country, the lower the usage of motorcycles, although this relationship is not strictly linear [3,4].

Human factors associated with motorcycle accidents may result from postural fatigue experienced during riding. Such fatigue may be caused by various factors, including damaged roads, bad weather conditions, inappropriate riding posture and mental load, overloaded payload, and high vibrations of the vehicle, among others [5,6,7]. High vibrations can be generated from several sources on the vehicle, such as the engine, drive train, low-quality tires, poor performance of the rear and/or front suspension, and incorrect positioning of feet and hands on the vehicle [8,9]. Additionally, the proximity of the driver and motorcycle enhances the direct impact of vibrations on the user. Therefore, assessing comfort in motorcycles is a crucial field of study, particularly in places where low-cost mobility options are necessary. It is an important variable for drivers, given the safety issues and subjective experience under different road conditions. Seat and feet vibrations have been identified as key factors contributing to discomfort, thus improving the ergonomics of these elements can significantly reduce driver fatigue and enhance safety. The suspension system plays a critical role in the overall performance of a motorcycle, particularly in terms of its ability to absorb vibrations and shocks resulting from the unevenness of the road surface. In this regard, researchers have mainly focused on ergonomics, semi-active suspension systems, and vibration absorption or isolation [5,10,11,12,13,14,15].

Comfort can be defined as a personal physical experience associated with a relaxed and less stressful state, while discomfort arises from biomechanical factors such as joint angles, postures, stresses, muscular contractions, and pressure distribution while seated [7]. A direct relationship exists between comfort and motorcycle vibrations, which are linked to the effects of vibrations on certain parts of the human body [16,17]. Vibrations have two fundamental effects, namely physiological and physical. In the former case, consequences may include an increase in heart rate or muscle tension; in the latter, there may be observed alterations in the vertebral column (backbone injury), degenerative processes of the lumbar segments, arthrosis, and issues with the digestive, genital, and urinary systems [10]. Harmful effects occur when vibrations reach the natural frequency of the human body, i.e., between 3 and 7 Hz. Furthermore, the human trunk has a resonance frequency between 4 and 8 Hz, with 4.4 Hz being the resonance frequency of lumbar vertebrae [18]. Thus, vibration transmission to these parts of the body, particularly vertical accelerations, may result in tissue failure, leading to continuous discomfort or pain in the lower back.

Two-wheeled vehicles are particularly sensitive to road conditions, such as roughness or uneven surfaces. The vibration spectrum produced by motorcycles typically ranges from 0.25 Hz to 20 Hz. Vibrations outside of this range either cannot be transmitted to the human body (if they are below 0.25 Hz) or are considered as noise (if they are above 20 Hz, as the vibration source is not the vehicle itself) [10]. The frequency and amplitude of accelerations determine the relative displacements and forces exerted on the driver’s body. In addition, the effects on the human body can increase depending on the length of exposure time and the pre-existing health conditions of the motorcycle rider. Additionally, the lower the smoothing of vibrations, the greater the maladjustment of the vehicle’s parts, leading to a shorter lifespan for the machine and higher costs for the owner.

The literature presents two primary approaches for assessing comfort in motorcycles: virtual and experimental testing. Virtual testing involves the use of computer-aided simulations to analyze the impact of vibrations on driver’s comfort [19,20,21,22]. However, this approach assumes an ideal vehicle performance, which is often not the case due to road conditions, maintenance issues, and physical modifications of the vehicle. Consequently, it can be difficult to assess comfort in low-cylinder engine motorcycles in developing countries using virtual environments, which makes the experimental comfort analysis a crucial process in comfort evaluation. While structural health monitoring (SHM) is a well-established field of study to evaluate vibrations in several structures [23,24,25], its application in the context of low-cylinder motorcycles is still in its early stages. Despite its importance, only a few studies have been carried out to address this issue [6,8,11,26]. In particular, the ISO 2631 standard provides a guideline to study the performance of vehicles and machines in relation to vibrations. Additionally, it provides a methodology to assess and establish limits for whole-body vibrations. Notwithstanding, more research is needed to experimentally analyze the comfort of motorcycles with different suspension system settings while running on real streets. Moreover, the instrumentation commonly used for this kind of testing is complex and often too expensive, making it difficult to perform affordable out-of-lab tests with practical applications.

The rear suspension damping in motorcycles is typically adjusted using a circlip that exerts preload on the suspension system’s spring. However, the appropriate setting depends on the available positions, the experience of the personnel, and subjective driver analysis. Therefore, an objective assessment is desirable when adjusting the suspension system. In this study, our aim is to experimentally analyze a low-cylinder engine motorcycle that is commonly used in low-income countries such as Colombia. We have designed a simple yet effective method by using a single triaxial accelerometer to first examine the vertical vibrations and later analyze the vibrations in all three directions detected by the sensor. We used a 125-cc engine motorcycle and varied the preload of the spring of the rear suspension system. For each preload condition, we evaluated the vertical acceleration using Vibration Dose Values (VDV) and Kurtosis metrics, which are widely used to analyze comfort in vehicles. To vary the input disturbance to the vehicle, we changed the height of speed bumps under controlled tests. This allowed us to analyze the real effect of the spring preload and determine its added value in a typical low-cost motorcycle. Finally, we conducted a comfort test over a non-controlled closed path with a length of 1.9 km.

## 2. Materials and Methods

### 2.1. Experimental Setup

**Figure 1:**
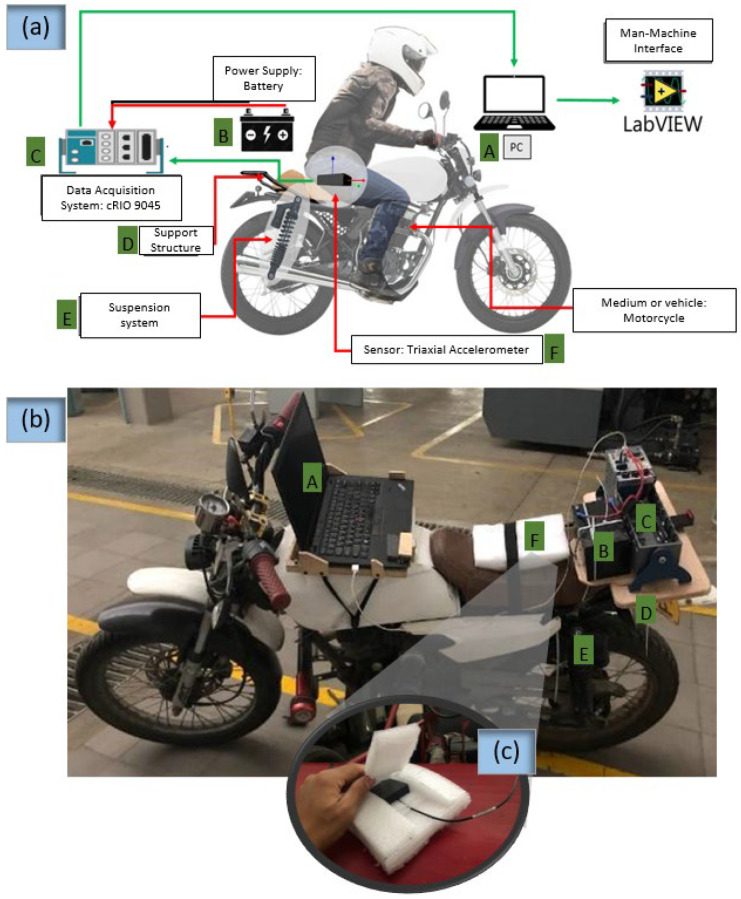
Implementation carried out to test comfort in a low-cylinder engine motorcycle. (**a**): sketch of the measurement chain used to analyze vibrations at the seat; (**b**): picture of the actual implementation; (**c**): housing for accelerometer sensor. Labels: A, PC and LabVIEW implementation; B, power supply; C, data acquisition system; D, mechanical support for the elements of measurement chain; E, rear suspension system; F, triaxial accelerometer.

Figure 1 depicts the experimental setup employed in this study. A triaxial accelerometer (B&K 4529-B, Bruel and Kjaer, Virum, Denmark) was mounted at the seat position, as illustrated in Figure 1a. To ensure reliable performance and reduce noise in the accelerometer signals, the sensor was enclosed in a small box designed according to the mechanical vibration and shock standard [27] (ISO 2631-1). The box was further covered with foam to prevent external disturbances, as shown in Figure 1c. The accelerometer was connected to a data acquisition system (cRIO-9045, National Instruments, Austin, TX, USA), and power was supplied by an electrochemical battery that only energized the data acquisition card. Data digitization was carried out using a laptop connected to the system through a LabVIEW interface. A picture of this setup is presented in Figure 1b. The motorcycle (NKD, 125 cc, Bajaj, Urana, India) used in the experiment was equipped with a CGR 4T OHV engine, manual gearbox, and maximum power and torque of 10.34 HP at 8000 RPM and 9.3 Nm at 7000 RPM, respectively. The original rear suspension system of the NKD 125 motorcycle was found to be faulty, prompting the decision to replace it with a comparable alternative taken from a Pulsar 180 motorcycle (Bajaj, India), and this replacement did not significantly alter the original behavior of the motorcycle.

**Figure 2:**
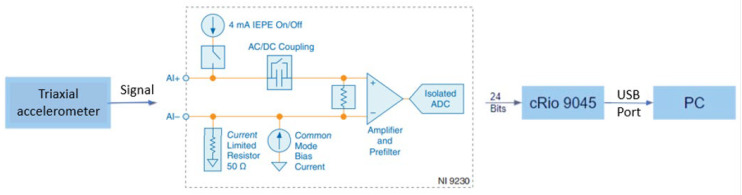
Block diagram that represents the digitization of acceleration data.

**Figure 3:**
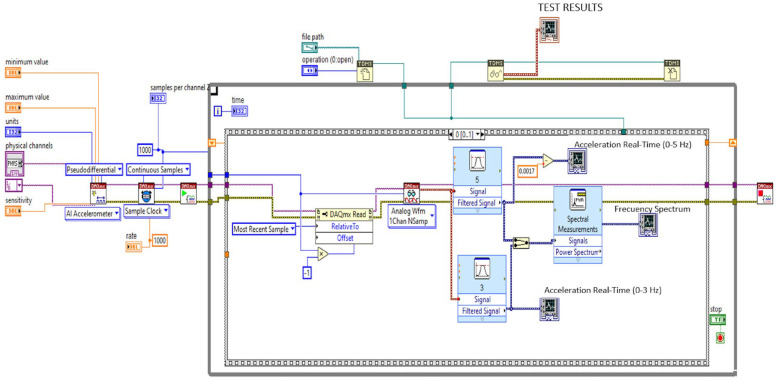
LabVIEW implementation to carry out the data acquisition using the cRIO-9045 system.

Figure 2 depicts the block diagram of the instrumentation stage used in this study. The triaxial accelerometer was linked to a data acquisition module (NI9230, National Instruments, Austin, TX, USA), which was installed on the cRIO-9045 rack. A LabVIEW interface was used to control this system via the laptop (refer to Figure 3). In order to ensure safety, we designed a framework to secure the laptop, external battery, and data acquisition system (shown in Figure 1b). The additional mass resulting from this equipment was below 6 kg, which accounts for less than 5% of the total weight of the motorcycle. Therefore, it did not have a significant impact on the vehicle’s vibration pattern.

### 2.2. Theory and Data Processing

#### 2.2.1. Mass–Spring-Dashpot Effective Model

**Figure 4:**
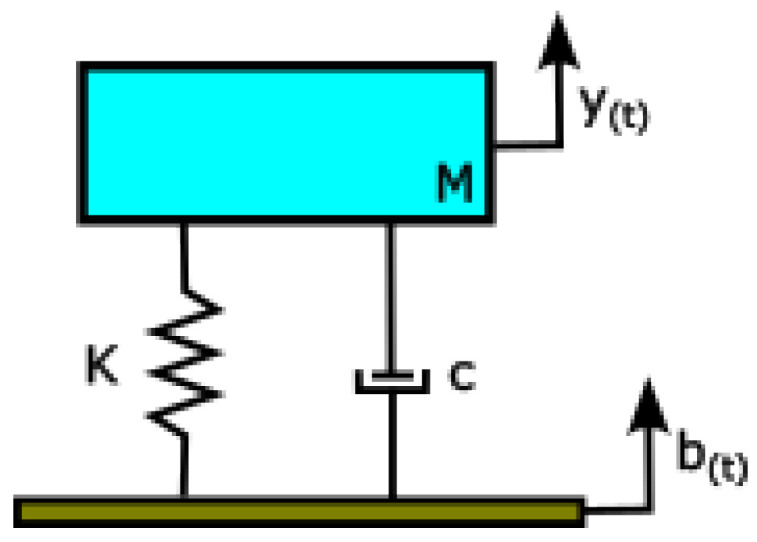
Sketch of a mass-spring-dashpot system. **M**: effective mass; **K**: effective stiffness; **c**: effective dashpot constant. Variables **b_(t)_** and **y_(t)_** represent the input and output displacements, respectively.

Figure 4 shows the effective model of a mass-spring-dashpot system. In this representation, the mass, M, encompasses the combined masses of the motorcycle, driver, and data acquisition system. The stiffness, K, primarily arises from the rear suspension system which varies with the chosen spring preload. The dashpot constant, c, accounts for the damping effect within the suspension system. Since the road condition is not constant due to the presence of bumps, we have included such effect by the input parameter, b(t). Lastly, the response of the system observed at the seat position is denoted as y(t).

The dynamic equation for the system of Figure 4 is

(1) $$M\ddot{y}+c\dot{y}+Ky=c\dot{b}+Kb$$

After application of the Laplace transform to (1), we obtain the transfer function of the system:

(2) $$\frac{Y_{\left(s\right)}}{B_{\left(s\right)}}=\frac{cs+K}{ms^{2}+cs+K}$$

where s, is the frequency variable in Laplace domain.

Next, we obtain the roots, $s_{1}$ and $s_{2}$, of the denominator of (2):

(3) $$s_{1},s_{2}=-\frac{c}{2M}\;\pm \;j\sqrt{{\frac{K}{M}-\left(\frac{c}{2M}\right)}^{2}}$$

where $j=\sqrt{-1}$, is the complex variable.

We can define the damping factor, α, and the natural frequency of the system, $\omega_{n}$, as follows:

(4) $$\alpha =\frac{c}{2M}$$

(5) $$\omega_{n}=\sqrt{\frac{K}{M}}$$

Thus, (3) can be rewritten as

(6) $$s_{1},s_{2}=-\alpha \pm {j\omega }_{n}\sqrt{1-{\left(\frac{\alpha }{\omega_{n}}\right)}^{2}}$$

The ratio in parenthesis is known as the damping ratio, $\zeta =\frac{\alpha }{\omega_{n}}$, which allows for the definition of the amplitude ratio at resonance, *A*, as follows:

(7) $$A=\frac{1}{2\zeta }=\frac{\omega_{n}}{2\alpha }=\frac{\sqrt{MK}}{c}$$

Consequently, the higher the effective stiffness, the higher the amplitude of the output displacement. Therefore, as the spring preload is increased, the vibrations transmitted to the seat position are amplified, resulting in a greater perception of discomfort by passengers.

#### 2.2.2. Signal Processing and Comfort Assessment

Acquired data were stored in a tridimensional matrix with dimensions of 6000 × 3 × 3 (time × bump height × spring preload). We removed the offset from signals and applied a low-pass filter with a cut frequency of 200 Hz (Butterworth, order 5) to eliminate external noise. To remove any further external influence, we windowed each signal using a Tukey window with a shape parameter set to 0.75 for 3000 samples. To perform time–domain analysis, we included the envelope of signals. To accomplish this, we obtained the analytical signal using the Hilbert transform and calculated its magnitude [28]. Steady-state analysis was carried out through the fast Fourier transform (FFT) to determine the main frequency content generated by the impacts with the bumps. Additionally, we used a time–frequency analysis to determine the contribution of each part of the path to the data information. This way, we defined temporal windows with their respective spectral content and identified the part of the signal that must be transformed to the Fourier domain. The time–frequency representation was obtained using the short-time Fourier transform (STFT), employing a Hann window, a segment length of 128 samples, 120 overlapping points, and a FFT length equal to eight times the number of samples in the segment.

To comprehensively analyze the shocks or impulsive velocity changes, specifically the presence of steep accelerations, we have utilized metrics based on the ISO 2631 norm, namely, the Vibration Dose Values (VDV) and Kurtosis. The VDV provides a suitable parameter for assessing vibrations by considering the magnitude, frequency, and exposure duration [29]. On the other hand, Kurtosis represents the fourth statistical moment of the signal and is highly sensitive to the impulsiveness of the time–domain data.

The VDV parameter is defined as follows [30]:

(8) $$VDV={\left[\int_{t_{1}}^{t_{2}}{\left[a_{w(t)}\right]}^{4}\right]}^{1/4}dt$$

where *t*_1_ and *t*_2_ define the temporal window of analysis, and $a_{w(t)}$ is known as the root-mean-square-frequency-weighted acceleration, which is expressed as:

(9) $$a_{w(t)}=\sqrt{\frac{1}{t_{2}-t_{1}}\int_{t_{1}}^{t_{2}}{\left[a_{\left(t\right)}\right]}^{2}dt}$$

where $a_{\left(t\right)}$ is the instantaneous vertical acceleration captured with the accelerometer.

To compute $a_{w(t)}$ and VDV, signals were numerically integrated using the composite trapezoidal rule. To estimate the instantaneous $a_{w}$, i.e., its value at each timestamp, we used the STFT transform and the Parseval’s theorem:

(10) $$\int_{-\infty }^{\infty }{\left|x_{\left(t\right)}\right|}^{2}dt=\int_{-\infty }^{\infty }{\left|X_{\left(f\right)}\right|}^{2}df$$

where $x_{\left(t\right)}$ and $X_{\left(f\right)}$ are the time and frequency representation of signal x, respectively.

Therefore, the integration of (9) was numerically computed along the frequency axis of the time–frequency distribution. This yielded an instantaneous representation for $a_{w(t)}$ for each *spring* preload and bump height, resulting in a tridimensional matrix. Then, by using (8), a VDV (a bidimensional matrix) was obtained for each case.

The time required to achieve the level of severe discomfort can be estimated as [29]:

(11) $$T_{15}={\left(\frac{15}{VDV}\right)}^{4}t$$

where t is the time over which the *VDV* was estimated.

The total *VDV*, regarding the three spatial components can be estimated:

(12) $${VDV}_{total}=\sqrt[{4}]{{VDV}_{x}^{4}+{VDV}_{y}^{4}+{VDV}_{z}^{4}}$$

On the other hand, Kurtosis was computed as [29]:

(13) $$K=\frac{1}{n\sigma^{4}}\sum_{m=1}^{n}{\left(a_{m}-\bar{a}\right)}^{4}$$

where a is the instantaneous acceleration detected by the accelerometer, $\bar{a}$ and σ are the mean value and standard deviation of a, respectively, and n the number of samples of the discrete signal.

### 2.3. Testing Procedure

#### 2.3.1. Comfort Assay under Controlled Testing

**Figure 5:**
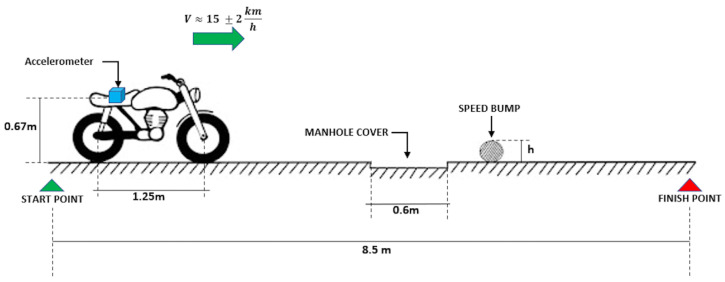
Sketch of the testing path. The preload of the rear spring is set among three levels: smooth, medium, and strong. The height of speed bump, h, changes in three levels: 25.4 mm, 38.1 mm, and 50.8 mm (the pipes are above the road). The unevenness of manhole cover respective to the floor level is negligible.

The experiments were conducted on an actual flat road under controlled conditions, as shown in Figure 5. The road surface was very smooth with negligible unevenness, and any minor sagging of the manhole cover was also disregarded. Only the vertical acceleration data in the z-direction of the sensor was analyzed to evaluate the suspension performance. In addition, no subjective analysis was carried out. To simulate the effect of road bumps on the vehicle, PVC pipes were used, and two variables were modified during the testing: the height of the bump (i.e., the diameter of the pipes) and the preload of the rear spring. Three different heights of 1″ (25.4 mm), 1.5″ (38.1 mm), and 2″ (50.8 mm) were used to simulate road bumps of different heights commonly encountered in Colombia. Typical heights on the street range between 20 and 80 mm [31]. The pipes were firmly fixed to avoid any misalignment during the testing.

**Figure 6:**
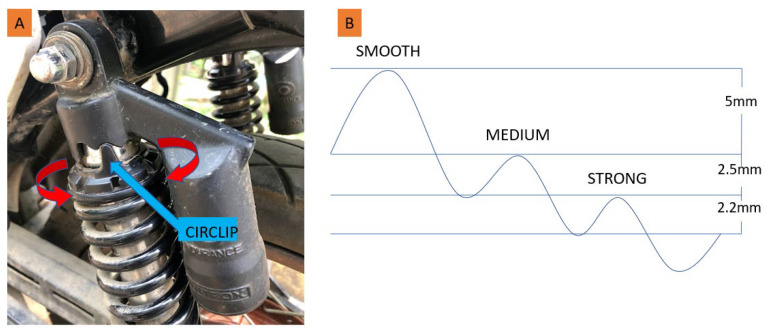
Adjustment of spring preload. (**A**) picture of the circlip mechanism. The arrows indicate the direction of increasing preload. (**B**) Preload levels in terms of displacement in the spring. The amplitude of the waves provides a visual representation of the oscillation achieved in each preload setting, serving as an indicator of the magnitude of the vibrations experienced.

The preload of the rear spring was set at three levels—smooth, medium, and strong—by adjusting a circlip on the motorcycle. Figure 6 depicts the circlip mechanism and illustrates the spring preload in relation to the displacement of the rear suspension system. While we did not measure the spring stiffness directly, we determined the compression of the spring at each preload setting: 5 mm for the smooth preload, 7.5 mm for the medium preload, and 9.7 mm for the strong preload.

The speed of the vehicle was controlled by the driver and set to 15 km/h ± 2 km/h. Each test was repeated three times for every possible combination of bump height and spring preload. The average behavior of the repetitions was stored to obtain a representative dataset for analysis and avoid random disturbances between measurements, i.e., we made an average of three repetitions for each possible combination of bump height and spring preload. The testing sequence is as follows: firstly, the acquisition system is activated while the motorcycle is started but stationary (the initial resting stage); subsequently, the vehicle is driven along the path illustrated in Figure 5 (the active stage); upon reaching the “finish point”, the motorcycle comes to a halt; and after this, the driver turns off the data acquisition (the final resting stage). The signals were sampled at a frequency of 1024 Hz and 6000 samples were stored for each measurement. This results in a total acquisition time of 5.9 s per test, encompassing both the resting and active stages. However, the active stage accounts for approximately 2 s of the total duration, which defines the window used for analyzing the experimental results. Since the testing time remains consistent across all tests, we can draw conclusions about the effects of the spring preload and bump height.

#### 2.3.2. Comfort Testing in a Closed Path (Non-Controlled Test)

**Figure 7:**
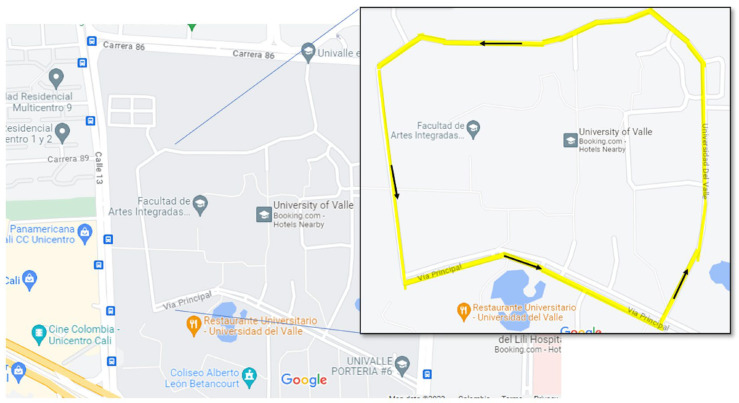
Comfort test in a closed path. The map represents the closed road inside Universidad del Valle’s campus.

We conducted the testing on a closed path within the campus of Universidad del Valle, located in Cali, Colombia. The total length of the path was 1.9 km, and it took approximately five minutes to complete (refer to Figure 7). The path encompassed various scenarios, including rough roads and speed bumps with different heights and slopes, making it an ideal route for assessing general comfort that closely resembles a real road experience. In this test, we collected data from all three axes of the accelerometer, but we did not conduct any subjective analysis. The average motorcycle speed was 24 km/h ± 1.5 km/h throughout the path, complying with Colombian law, which requires vehicle speeds to be no more than 30 km/h in gated communities that are frequented by pedestrians. We conducted two tests, each with a spring preload set at the two extreme positions, i.e., the smooth and strong preloads (see Figure 6). Both measurements were processed using the ISO 2631 filtering method for weighted accelerations, enabling us to obtain a global quantification of passenger comfort. While this standard does not prescribe a specific test duration, it emphasizes the importance of gathering sufficient data to draw meaningful conclusions. Therefore, we selected a test duration close to five minutes to fulfill this requirement. In these conditions, evaluating comfort requires considering all three directions. Therefore, we recorded the accelerations $a_{x}$, $a_{y}$, and $a_{z}$, and subsequently, we applied (8) to calculate the total VDV.

## 3. Results

### 3.1. Analysis of Acceleration Signals

**Figure 8:**
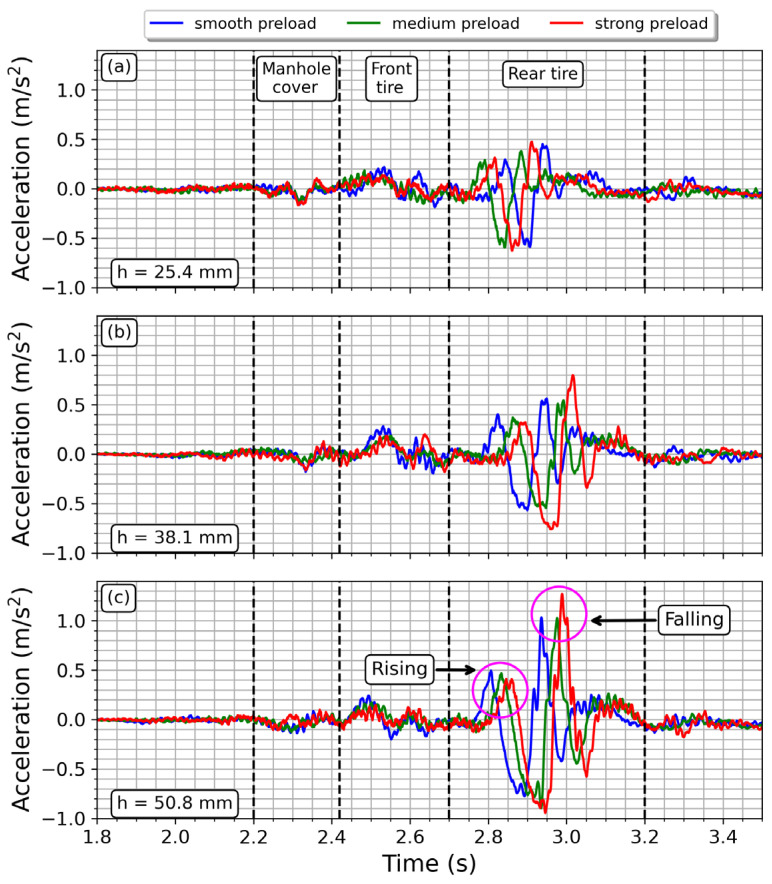
Averaged time–domain signals obtained when varying the spring preload and height of the speed bump. (**a**) small height, (**b**) mid height, (**c**) greater height. The vertical discontinuous lines indicate specific time windows for analysis. Significant acceleration is observed at the seat position when the rear suspension is working. In subplot (**c**), labels have been added to denote the zone where the rear tire impacts the bump; the first and second maxima in the signal correspond to the instances when the rear tire is entering (rising) or leaving (falling) the bump, respectively. This is also valid for subplots (**a**,**b**).

Figure 8 displays the nine signals obtained from the tests conducted, which are divided into three subplots that represent each speed bump height. The initial time is the same for all the signals. The vertical discontinuous lines on the plot indicate the stages of the driving path as the motorcycle moves forward. The first vibration detected by the sensor is generated by the front tire impact with the bump, but it is smaller compared to the vibrations due to the rear tire. The stage labeled as “manhole cover” represents the signal captured when the rear tire gets onto the cover. In this case, the front tire does not generate a significant vibration at the seat position before 2.2 s (see Figure 8). When the motorcycle arrives at the bump, the front tire strikes the obstacle, generating the first vibration at the seat position. This is labeled in Figure 8 as “front tire”, next to it is the signal detected when the “rear tire” impinges the bump. The differences in time of signal peaks are due to the challenge of maintaining a specific velocity while driving. However, such differences were less than 0.2 s, which is small considering that speed control relied on the average driver’s ability. The rear tire stage highlights that the rear suspension is more critical than the front suspension for the driver’s health. The higher the height of the speed bump, the higher the acceleration at the seat position. Furthermore, the acceleration increases when the preload of the spring is increased, which agrees with what is expected from (7).

**Figure 9:**
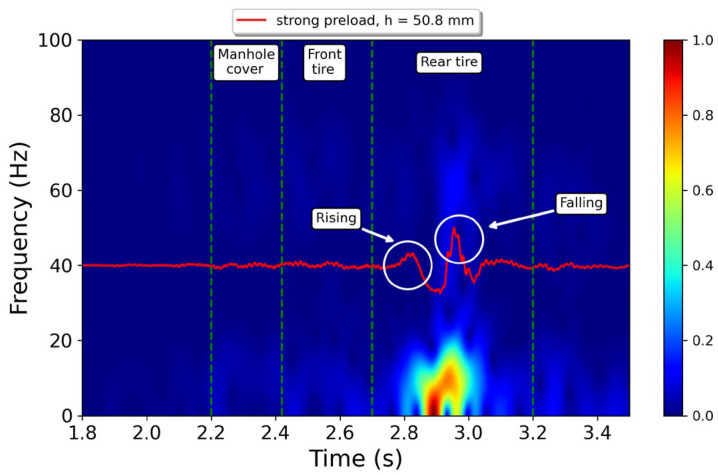
Time–frequency representation of a signal detected by the accelerometer when the rear spring was set to strongest position and the bump height was 50.8 mm. The colorbar is normalized. The vertical discontinuous lines represent the time windows when the motorcycle traverses either the manhole cover or the speed bump with its front/rear tires. The labels “rising” and “falling” indicate the specific moments when the rear tire enters or leaves the bump, respectively.

At the bump, the rear suspension displays a distinct response when the tire is rising or falling over the obstacle. This behavior has been labeled in Figure 8c. During the rising stage, the acceleration produced exhibits a single maximum peak followed by a single decay. On the other hand, during the falling stage, the acceleration shows the highest peak of the signal followed by one or two smaller peaks due to damping. A similar behavior is observed in Figure 8a,b. Figure 9 illustrates the time–frequency distribution obtained, using the STFT, of an acceleration signal captured by the sensor in the vertical direction. This specific measurement was taken with the rear suspension system adjusted to the highest preload setting and with the tallest bump in the road. The spectral information has maximum intensity during the rising stage, whilst the higher frequency content is observed when the rear tire is falling. Therefore, the most substantial input to the driver’s backbone occurs when the motorcycle is leaving the bump, which is the moment when the driver experiences the most discomfort.

**Figure 10:**
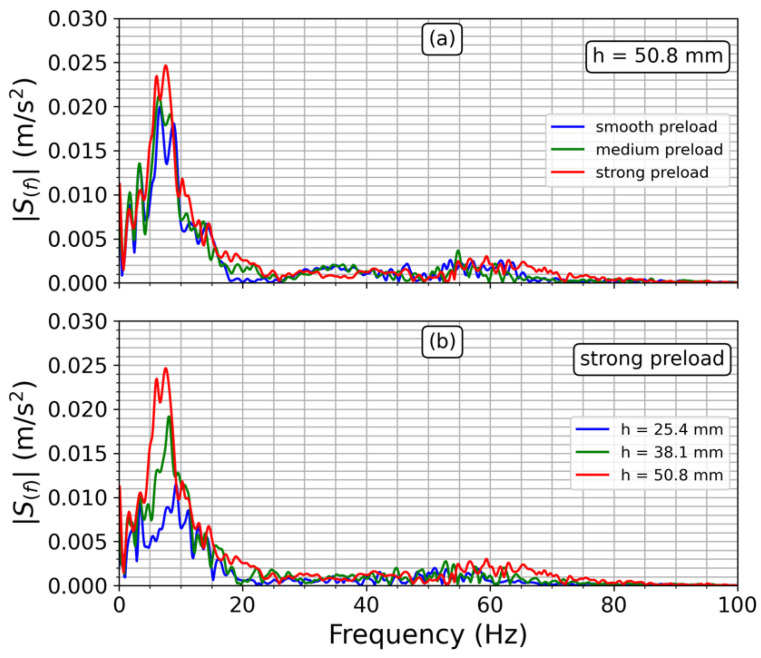
Frequency–domain representation of acceleration signals. The vertical axis in (**a**,**b**) represents the magnitude of the acceleration signals. (**a**) preload analysis with the higher bump height. (**b**) bump height effect when spring preload is the strongest.

Figure 10 depicts the results of a steady-state analysis conducted in the Fourier domain. The findings indicate that increasing the preload and bump height leads to higher vibration intensity, i.e., discomfort and potential backbone injuries. The signals’ bandwidth includes the natural frequency of the human body [18], with our experimental results indicating that the primary spectrum energy is between 5 and 10 Hz. Therefore, particular attention should be paid to the rear suspension, especially when traversing poor quality roads or rural paths that feature roughness of tracks, uneven or wavy surfaces, and holes amid the streets. Furthermore, the higher the bump height at the same spring preload, the wider the vibration bandwidth and intensity, resulting in a more uncomfortable and hazardous driving experience (refer to Figure 10b). Additionally, increasing the preload results in more frequency components being added to the acceleration signals, as evidenced by Figure 10a and Equation (6).

**Figure 11:**
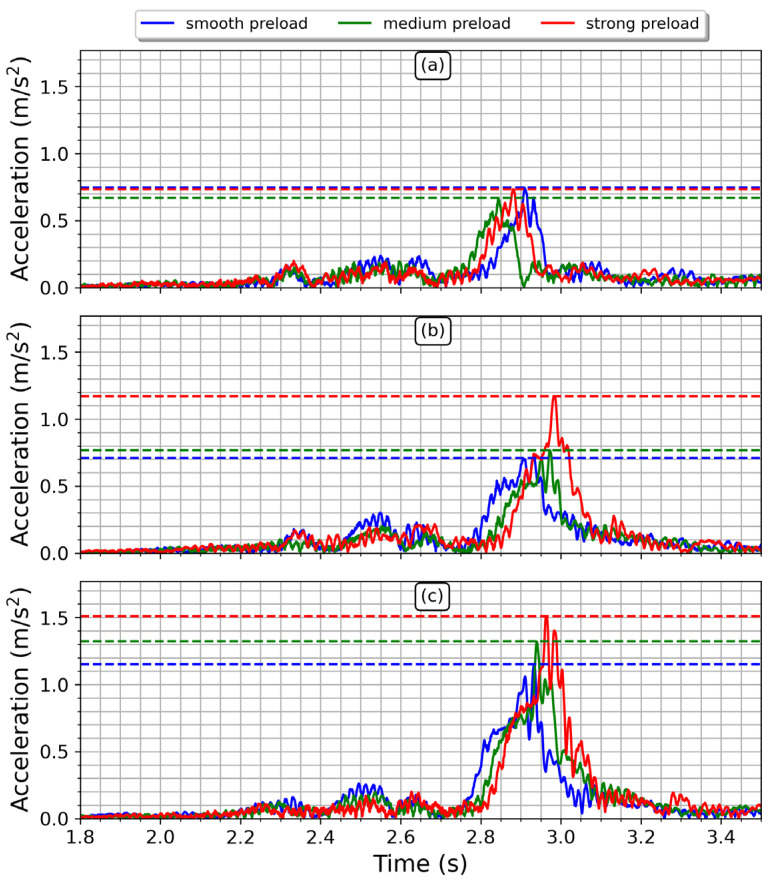
Envelope of acceleration signals captured when spring preload and bump heights were changed. (**a**) h = 25.4 mm, (**b**) h = 38.1 mm, (**c**) h = 50.8 mm.

Figure 11 plots the envelope of the signals drawn in Figure 8. When the speed bump is small, the effect of the spring preload is negligible, meaning that vibrations are transmitted to the seat equally, irrespective of the preload settings. Therefore, when street roughness is small, the preload effects are insignificant. However, for an intermediate bump height of 38.1 mm, the preload becomes significant, especially for the extremes of smooth and strong preloads. On the other hand, the behavior of the medium preload setting is almost the same as that of the smooth preload setting. Finally, when the bump height reaches its maximum value of 50.8 mm, the spring preload setting becomes distinguishable, and the driver will perceive vibrations differently depending on the rear suspension setting. In addition, the higher the bump height, the greater the vibrations perceived by the user, regardless of the spring preload setting. Moreover, the vibration transmitted to the user increases as the stiffness of the suspension system increases, as expected from (7).

### 3.2. Comfort Analysis under Controlled Driving Conditions

**Figure 12:**
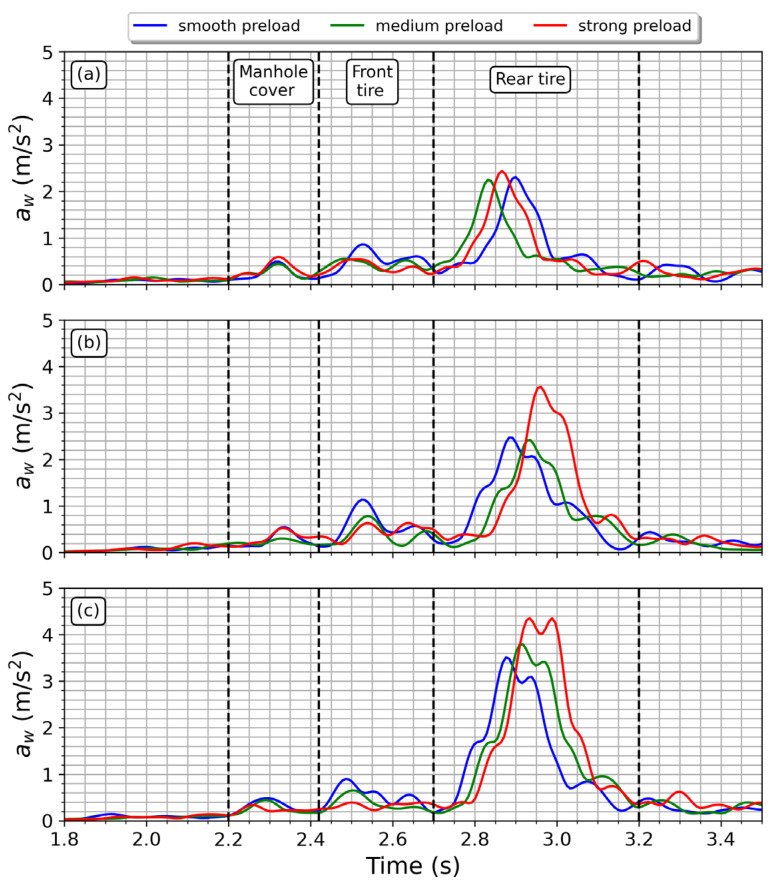
Frequency-weighted root mean square acceleration obtained when spring preload and bump heights (h) were changed. (**a**) h = 25.4 mm, (**b**) h = 38.1 mm, (**c**) 50.8 mm. Vertical discontinuous lines represent the time windows when the motorcycle traverses either the manhole cover or the speed bump with its front/rear tires.

Figure 12 shows the root-mean-square-frequency-weighted acceleration, a_w_, computed by application of (9). It can be seen that the rear tire zone is the most significant moment for vibration transmission. The contribution of each preload setting to a_w_ agrees with the behavior observed in Figure 11.

**Figure 13:**
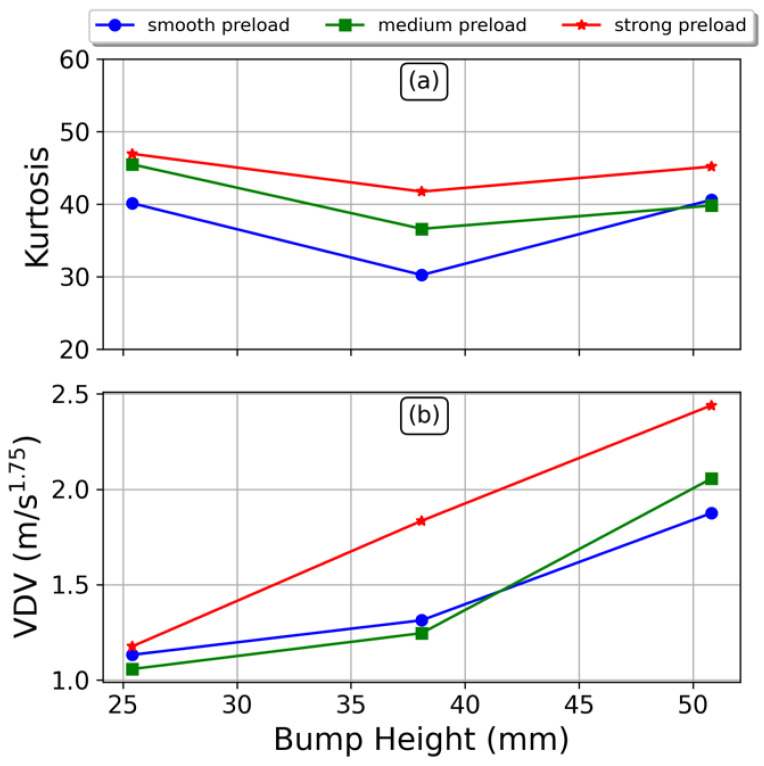
Kurtosis, (**a**), and Vibration Dose Values or VDV, (**b**), of acceleration signals.

Figure 13 contains the behavior of the Kurtosis, (a), and VDV, (b). Both parameters provide absolute values accordingly to Equations (8) and (13). We have disaggregated the effects of spring preload (line colors) and the bump height (horizontal axis), such that we can infer their impact in the passenger comfort. The Kurtosis provides insights into the degree of impulsiveness, and consequently, the magnitude of the impact transmitted to the seating position. On the other hand, the VDV enables the determination of the level of discomfort experienced by the passenger. It is observed that increasing the preload results in a higher perceived impact by the driver. However, it is noteworthy that the discomfort level remains relatively consistent when the preload is set to the two lower positions. While the transmitted impulse to the passenger increases gradually with higher preload (see Figure 13a), the effect on perceived comfort is only significant at the highest preload due to the damping mechanism (see Figure 13b). This suggests that, in addition to the preload, the damping plays a crucial role in mitigating the impact and maintaining a consistent level of comfort, especially for lower preloads, as can be inferred from (6).

Values for kurtosis in Figure 13a range between 5 and 10, which implies that data contain several extreme values (this is also confirmed in Figure 8). Notwithstanding, the impulsiveness is the same regardless the spring preload, i.e., the impact received at the seat is the same without significant difference given the specific rear suspension setting. Table 1 contains the mean and standard deviation, at specific positions, of Kurtosis and VDV values in Figure 13. From Figure 13b, the higher the bump height the higher the VDV, hence, the discomfort increases. At the lower height the measured vibrations are pretty much the same regardless the preload in spring (see Figure 13a and Table 1). At half height, the smooth and medium preloads are quite similar, while the strong preload separates providing more vibrations at the seat, which explains the high standard deviation observed in Table 1 at h = 38.1 mm. Finally, at the taller bump the three preloads are distinctive. This is also observed in Table 1. This behavior agrees with results of Figure 11, showing that acceleration envelope, or its corresponding area, can be used as a metric for comfort analysis too.

By application of Equation (11), using a VDV time equal to 0.5, at the smooth preload the time required for severe discomfort is close to 5 h, while at the strong preload it is 20 min. At the medium preload, it is a little more than an hour. Therefore, regarding the whole-body vibrations, the lower severity is obtained with the smooth preload, though the lesser the stiffness the higher the oscillations of the seat.

### 3.3. Comfort Testing through an Uncontrolled Closed Path

The comfort tests conducted on the closed path lasted approximately five minutes, and the results are summarized in Table 2. For this test, we set the preload at two extreme positions, namely, smooth and strong. The results indicate that in the former, the time to severe discomfort is over one hour, while in the latter, it is close to half an hour. These findings highlight the importance of paying careful attention to vehicle performance, such as preventive maintenance, regardless of the preloads. Considering that traffic in a crowded city typically takes over one and a half hours to reach the destination, it is imperative to subject a motorcycle with the characteristics observed in our test vehicle to a thorough inspection. It is evident that neither the smooth nor the strong preloads guarantee healthy use.

## 4. Discussion and Conclusions

In this study, we have developed a characterization system utilizing a triaxial accelerometer to investigate the comfort level of a low-cylinder engine motorcycle. The system provides a means to quantify driver fatigue and pain, which can aid in the establishment of exposure thresholds and policy development to ensure driver safety. Our approach adheres to ISO-2361 guidelines for evaluating Vibration Dose Values (VDV) and Kurtosis in the motorcycle under varied experimental conditions. Specifically, we varied the rear suspension system preload and subjected the vehicle to impulse inputs via speed bumps of different heights. Additionally, we conducted a real-world evaluation of the motorcycle’s comfort level around a closed path in the campus of Universidad del Valle in Cali, Colombia.

We found that when encountering bumps as low as 25.4 mm, the transmission of vibrations in the motorcycle is equal regardless of the preload setting of the rear suspension system. However, when bump height increases, vibration at the seat position becomes distinguishable. The higher the spring preload, the greater the vibration transmission, resulting in increased discomfort for the driver. This observation agrees with the expected behavior predicted by the effective model of a mass-spring-dashpot system, as can be seen from (7). This finding suggests that a simple model can serve as a rapid and uncomplicated tool for setting the performance parameters of a motorcycle intended for daily use. Additionally, the descending stage of the bump is more critical than the ascending stage, producing higher acceleration peaks at the seat. This increases the risk of backbone injury, particularly for frequent users such as delivery personnel or police officers. While we utilized conventional metrics such as the Vibration Dose Values (VDV) and Kurtosis to evaluate comfort in the motorcycle under study, we also confirmed that the envelope of the acceleration and the root mean square weighted acceleration provide information about the differences in preload setting and bump height. In fact, these curves provide more information than the scalars values obtained with VDV and Kurtosis: the acceleration curve or its envelope enable the determination of the stages or moments when vibrations are more critical and the vehicle part from which such vibrations originate. This aspect is of significant importance as both Kurtosis and VDV represent scalar values, that offer a comprehensive overview of comfort without explicitly indicating instances when impulses exceed permissible levels. Consequently, our work allows for a targeted approach towards preventive maintenance, specifically directing attention to components that are more susceptible to significant deviations in actual roads of low-income countries. In such regions, road conditions often deviate significantly from ideal conditions, rendering factory-design parameters inadequate for real-world scenarios. Therefore, it becomes crucial to evaluate the performance of motorcycles based on these actual conditions rather than relying solely on nominal design parameters.

In summary, this study presents a simple yet valuable implementation that can aid in developing preventive and corrective strategies to minimize drivers’ exposure to vibrations. Furthermore, it can provide quantitative measurements of a vehicle’s performance straight out of the garage, which is a fundamental issue in developing countries where vehicles tend to quickly fall out of adjustment on the road. The results presented in this work lead to the conclusion that vertical acceleration plays a crucial role in quickly estimating the comfort level and generating extensive data for statistical modeling in public health. While a comprehensive understanding of vehicle comfort necessitates three-dimensional data, this initial step focusing on a single axis can still offer valuable information for public use. Therefore, this approach provides crucial information on the suspension system behavior for personal care under real-world conditions, to identify risk factors related to drivers’ exposure to vibrations, passenger posture, vibration duration, and other ergonomic factors that affect personnel. A widespread adoption of portable systems with uniaxial accelerometer sensors could provide sufficient data to support the establishment of evidence-based public health policies and propose new standards for the transport industry based on out-of-lab measurements directly obtained from the road, rather than estimated with approximate models. Currently, we are conducting a performance evaluation of various vehicles, including electric bicycles, four-wheel automobiles and SUVs, with the aim of obtaining additional information on aspects such as comfort, efficiency, and stability. This evaluation entails an analysis of the interrelationship between these parameters and their effect on a passenger’s comfort.

## Figures and Tables

**Table 1:** Mean and standard deviation of kurtosis and VDV. Variable h represents the bump height.

	Kurtosis			VDV(m/s^1.75^)		
h (mm)	25.4	38.1	50.8	25.4	38.1	50.8
Mean	44.2	36.2	41.9	1.1	1.5	2.1
Standard deviation (%)	6.6	13.0	5.7	4.4	18.0	11.1

**Table 2:** VDV obtained for comfort analysis in the closed path.

Preload	VDV_total_ (m/s^1.75^)	T_15_ (min)	Proceeding
Smooth	7.6	75.9	Action must be taken if driving time is greater than 1 h
Strong	9.0	38.6	Action must be taken if driving time is greater than half an hour

## Data Availability

Data are available upon reasonable request.
